# Solitary Fibrous Tumor of the Prostate Which Was Initially Misdiagnosed as Prostate Cancer

**DOI:** 10.1155/2017/3594914

**Published:** 2017-02-26

**Authors:** Soma Osamu, Hiromi Murasawa, Atsushi Imai, Shingo Hatakeyama, Takahiro Yoneyama, Yasuhiro Hashimoto, Takuya Koie, Chikara Ohyama

**Affiliations:** Department of Urology, Hirosaki University Graduate School of Medicine, Hirosaki 036-8562, Japan

## Abstract

Solitary fibrous tumor (SFT) of the prostate is a very rare tumor. We report a case of 65-year-old man with SFT of the prostate which was initially misdiagnosed as prostate cancer. Finally, we performed total prostatectomy and the tumor was histologically diagnosed as SFT of the prostate. The patient's clinical course has progressed favorably with no obvious recurrence 18 months postoperatively.

## 1. Introduction

Solitary fibrous tumor (SFT) is a spindle-cell tumor derived from fibroblasts [1]. It preferentially occurs in the pleura and accounts for two-thirds of all cases [2]. Prostate SFT is extremely rare, with only approximately 20 cases reported to date [3]. Here we report an additional case of prostate SFT with an 18-month follow-up.

## 2. Case Report

The patient was a 65-year-old Japanese man who was examined by his family doctor for the chief complaint of nocturia. He was treated for benign prostatic hyperplasia, but the symptoms did not improve. Ultrasonography (US) and computed tomography (CT) were performed and an intrapelvic tumor was suspected. The patient was then referred to our department.

No definite abnormal findings were observed on blood biochemistry tests. His prostate-specific antigen level was within the normal range at 0.92 ng/mL. Values for other tumor markers also fell within the normal ranges. A giant tumor with a central, hypoechoic region was noted on the right side of the prostate during US.

**Figure 1:**
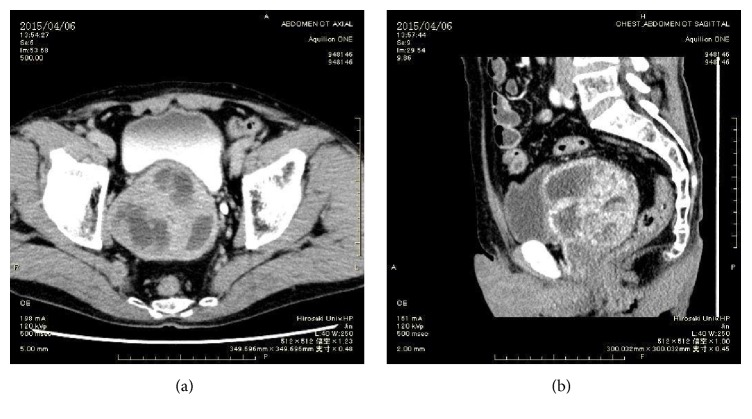
CT scan image of prostatic tumor. (a) Axial image. (b) Sagittal image.

**Figure 2:**
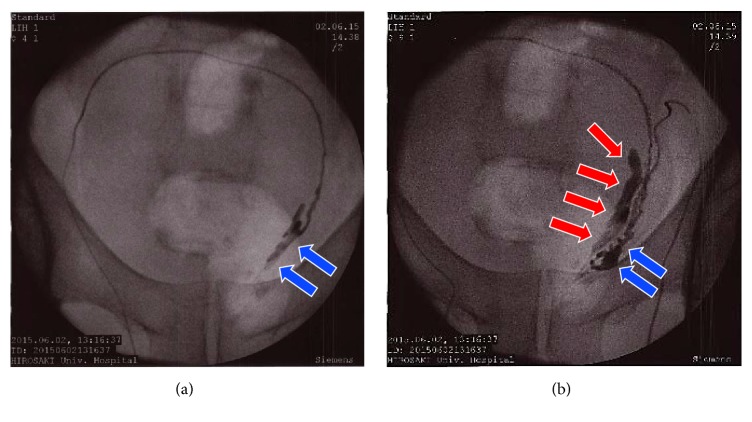
Image of vasography. (a) Right vasography; (b) left vasography. Blue arrow: right seminal vesicle. Red arrow: left seminal vesicle. Vasography showed that the right seminal vesicle was largely shifted to the left side but shape of right seminal vesicle was kept.

Contrast-enhanced CT showed a tumor measuring 100 mm along the major axis and mainly present from the right side of the prostate (Figure 1). The tumor was composed of solid and cystic components associated with contrast enhancement. We suspected the tumor originated from the right seminal vesicle. However, based on vasography examination, the right seminal vesicle was shifted to the left side, but its shape was maintained. We concluded that the tumor originated from the prostate (Figure 2).

**Figure 3:**
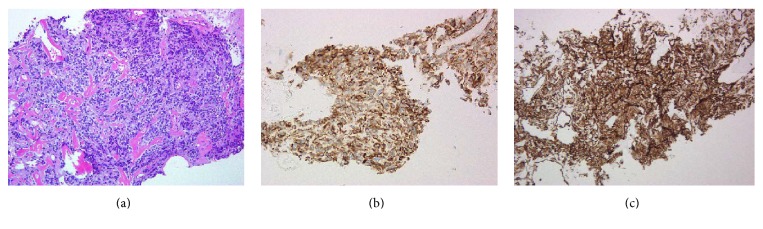
Microscopic findings of prostatic biopsy. (a) HE staining; (b) AE1/AE3; (c) vimentin (original magnification ×40).

We suspected infiltration of the bladder when we examined sagittal views. However, cystoscopy showed no obvious bladder infiltration, and the neck of the bladder was displaced to the right side by the tumor. A prostatic biopsy was performed in our hospital. Immunostaining was positive for both cytokeratin AE1/AE3 and vimentin. The patient was finally diagnosed with a poorly differentiated adenocarcinoma (Figure 3).

Based on the results of pathological examinations, the patient was diagnosed with cT4N0M0 prostatic cancer. We administered combined androgen blockade treatment and chemotherapy with docetaxel, but they were ineffective. Therefore, we discussed the options with the patient and decided to perform surgery. While discussing the surgical technique, we communicated viable options, such as total intrapelvic resection and total cystectomy. We explained that the decision would be made based on the intraoperative findings.

We decided to perform total prostatectomy. The mucosal surface of the bladder was maintained and the bladder was preserved. The tumor was continuous with the capsule on the right side of the prostate.

**Figure 4:**
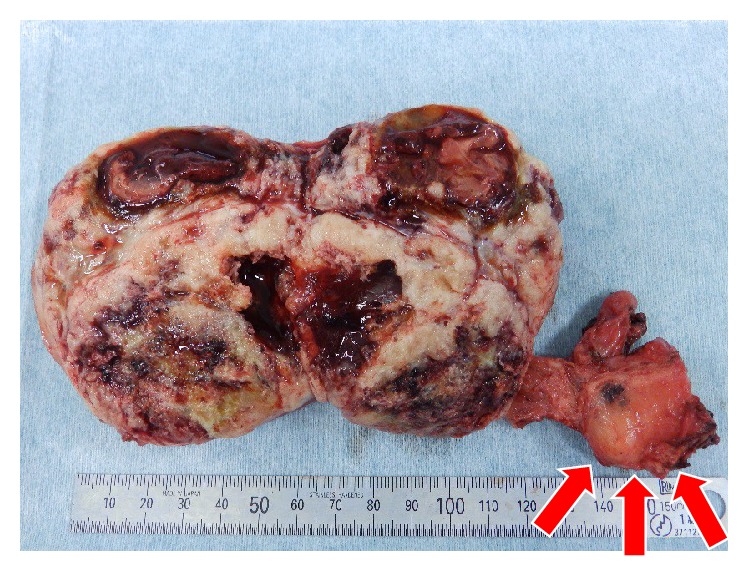
Macroscopic finding of resected prostatic tumor. Red arrow: prostate.

Macroscopically, we observed an oval-shaped tumor with a smooth surface and distinct margins that was continuous with the prostatic capsule (Figure 4). The resected surface was made up of solid components of a grayish white color, with areas of necrosis and hemorrhage.

**Figure 5:**
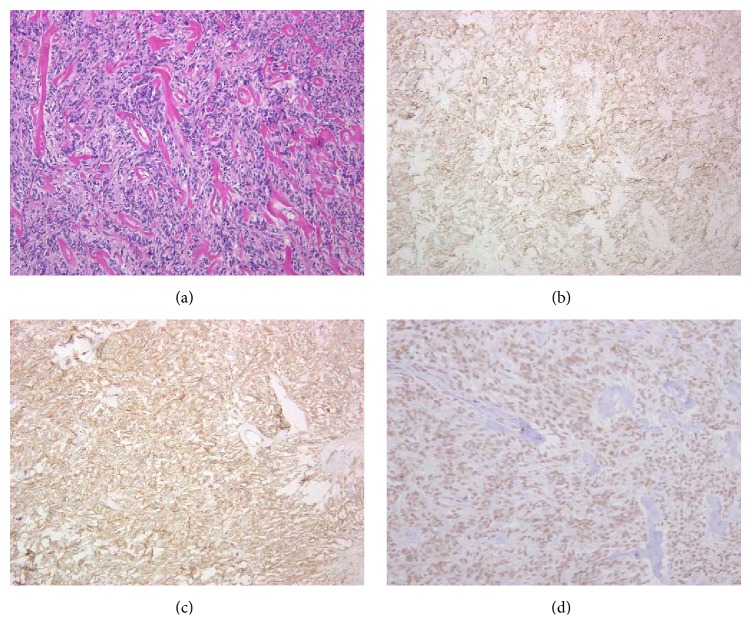
Microscopic finding of resected prostatic tumor. (a) HE staining; (b) AE1/AE3; (c) CD34; (d) STAT6 (original magnification ×100).

Histologically, we saw the proliferation of short spindle and polygonal cells on a background of abundant vessels showing vascular mural hyalinization (Figure 5).

Immunohistostaining was positive for cytokeratin AE1/AE3 and CD34.

Staining was negative for c-kit, desmin, *α*-SMA, and EMA, and the Ki 67 index was less than 2%. A definitive diagnosis was also difficult to achieve based on these immunostaining results. By consulting Dr. Hasegawa, the Director of Pathology at Sapporo Medical University, we reached a diagnosis of a solitary fibrous tumor, based on the fact that the tumor tested positive for CD34 and STAT6.

Postoperatively, there were no obvious complications and the patient was discharged. The patient's clinical course has progressed favorably with no obvious recurrence 18 months postoperatively.

## 3. Discussion

While SFT preferentially occurs in the pleura, it has also been reported to occur in other parts of the body [2]. It is a borderline malignant tumor, and 10%–20% of pleural SFTs are considered malignant [4]. Prostate SFT is extremely rare, with only approximately 20 cases reported to date [3].

A definitive diagnosis of SFT was difficult to establish in the present case for several reasons. First, a biopsy specimen was found to be positive for cytokeratin. Among various forms of the epithelial marker cytokeratin, AE1/AE3 is useful to distinguish between poorly differentiated/undifferentiated and mesenchymal cancers. Although this patient was clinically predicted to have mesenchymal tumor due to low PSA level, the biopsy finding of cytokeratin AE1/AE3 positivity was deemed importance, and the patient was diagnosed with poorly differentiated carcinoma. The possibility that an SFT is histologically positive for AE1/AE3, as observed in our patient, is supported by the literature, wherein AE1/AE3 positivity has been reported in 3 of 27 SFT patients [5]. Therefore, it does occur, albeit very rarely.

Second, SFT was not included in the differential diagnosis. Spindle-cell tumors pertinent to the prostate are listed as follows [6].

*Spindle Lesions of the Prostate*

1. Stromal tumors of uncertain malignant potential
2. Stromal sarcoma
3. Sarcomatoid carcinoma
4. Leiomyoma
5. Leiomyosarcoma
6. Rhabdomyosarcoma
7. Inflammatory myofibroblastic tumor
8. Gastrointestinal stromal tumor
9. Solitary fibrous tumor

They are discriminated based on histopathological findings and immunohistochemical staining results that are characteristic of specific types of tumors. In this case, the immunostaining tests conducted at the time of biopsy did not include markers consistent with SFT diagnosis, such as STAT6, CD34, and CD99 [3, 7, 8], and we believe that this made the definitive diagnosis difficult.

Below, we will discuss if the administered treatment was appropriate in the present course. Andrea et al. have recommended the following treatment plan for prostate SFT: (1) A repeat biopsy should be performed to achieve a more accurate diagnosis, (2) short-term follow-up is advisable for elderly patients with a small lesion, and (3) nerve-sparing radical prostatectomy should be performed in young patients with a large lesion and serious urinary tract symptoms; furthermore, cystoprostatectomy should be performed if invasion to the bladder is identified [6].

Nair et al. have emphasized that the complete removal of tumors is the most important predictor of prognosis in prostate SFT [9]. They discussed approximately 17 prostate SFT cases. Four patients had a positive margin, including one patient who had a recurrence 12 months after removal of the tumor and then underwent radical prostatectomy. Of the remaining 3 cases, 1 died of perioperative complications and 2 did not experience a recurrence. Thus, there was only 1 case of recurrence in a total of 17 cases, including negative margin cases [9]. In the present case, the surgical procedure was appropriate and the margin status was negative. Therefore, we believe that an appropriate treatment method was chosen.

In conclusion, we report an additional case of prostate SFT which was initially misdiagnosed as prostate cancer. Finally, we performed total prostatectomy and histologically diagnosed SFT of the prostate. When we encounter prostatic tumors histologically derived from spindle cells, we must consider SFT as one of the differential diagnoses.
